# Wound Healing Efficacy of Novel Dermal Substitute TissueDerm Graft^®^ in Full-Thickness Skin Defects

**DOI:** 10.3390/polym18151885

**Published:** 2026-07-31

**Authors:** Kyu-Sik Shim, Dohyun Kim, Jeongwoo Park, Han-Saem Jo, Nayeon Koo, Won Jai Lee, Tai Suk Roh, Wooyeol Baek

**Affiliations:** 1 PLCOskin Co., Ltd., Seoul 03721, Republic of Korea; sksbio@plcoskin.com (K.-S.S.); dohyun3343@plcoskin.com (D.K.); jwpark@plcoskin.com (J.P.); gkstoa0525@plcoskin.com (H.-S.J.); nykoo@plcoskin.com (N.K.); 2 Department of Plastic and Reconstructive Surgery, Institute for Human Tissue Restoration, Severance Hospital, Yonsei University College of Medicine, Seoul 03722, Republic of Korea; pswjlee@yuhs.ac (W.J.L.); rohts@yuhs.ac (T.S.R.)

**Keywords:** wound healing, skin tissue regeneration, skin grafting, collagen, full-thickness skin defect

## Abstract

Autologous skin grafting is considered the standard treatment for full-thickness skin defects and provides essential dermal reconstruction to support both functional and aesthetic skin outcomes. However, it has limitations, such as increased donor-site morbidity, suboptimal aesthetic outcomes, thickness mismatches, texture irregularities, and scar contractions. Several dermal substitutes have been developed to overcome these limitations. In the present study, we introduce TissueDerm Graft^®^, a novel collagen-based dermal substitute designed to improve both functional and aesthetic outcomes in skin grafting by providing a regular structure that supports dermal tissue regeneration. We compared the properties and efficacy of TissueDerm Graft^®^ with those of bovine acellular dermal matrix (Matriderm^®^). A full-thickness skin defect was induced in minipigs and TissueDerm Graft^®^ or Matriderm^®^ were simultaneously transplanted with a split-thickness skin graft (STSG). In the present study, TissueDerm Graft^®^ exhibited tightly packed and uniform collagen fibers, a consistent pore distribution, and elastin. The full-thickness skin defect model demonstrated favorable wound healing without inducing excessive inflammatory responses or excessive scar formation. Compared with the single implantation of STSG and co-implantation of STSG and Matriderm^®^, the co-implantation of STSG and TissueDerm Graft^®^ improved the visual outcomes. These findings suggest that TissueDerm Graft^®^ could serve as a promising dermal substitute for skin grafting and skin tissue regeneration.

## 1. Introduction

Skin defects frequently arise from various causes, including stab wounds, lacerations, infections, burns, trauma, and surgery, and present significant challenges for effective management. These wounds undergo a complex healing process, comprising hemostasis, inflammation, proliferation, and remodeling [1,2]. During these phases, reepithelialization is initiated by keratinocyte migration and stem cell differentiation, whereas collagen inflow—stimulated by growth factors from immune cells and fibroblasts—supports structural integrity [3]. However, in full-thickness burns or chronic wounds, natural healing processes are significantly impaired, requiring advanced therapeutic interventions to enhance wound repair [4,5,6,7].

Skin grafting is regarded as the standard treatment for full-thickness skin defects, providing essential dermal reconstruction to support both functional and aesthetic outcomes [8,9]. However, it has inherent limitations: full-thickness grafts and flaps, although potentially yielding better results, are associated with increased donor-site morbidity. Furthermore, skin grafts often lead to suboptimal aesthetic outcomes, characterized by thickness mismatches, texture irregularities, and scar contraction. These limitations underscore the need for additional strategies, such as dermal substitutes, to achieve optimal reconstruction quality [10,11,12].

To address these limitations, various skin substitutes have been developed to treat deep dermal and full-thickness wounds, offering a more natural dermal structure with enhanced reepithelialization [12,13,14,15], which is critical for wound healing, and occurs through the migration and proliferation of keratinocytes from the wound edges and the differentiation of stem cells from residual hair follicles [16,17,18]. Collagen is also crucial for providing structural support during wound repair. Collagen deposition during wound healing is promoted by growth factors secreted by macrophages, platelets, and fibroblasts, as well as by fibroblast proliferation and subsequent synthesis of the dermal matrix [19,20].

Numerous substitutes have been developed to improve healing outcomes. However, some have limitations, such as engraftment failure, delayed vascularization, high costs, and limited shelf life [21]. Therefore, collagen has been used as an alternative therapy. Collagen is the most abundant extracellular matrix (ECM) component in mammals and a major element of connective tissues, including the skin. Type I collagen promotes cell attachment, proliferation, and overall wound healing [19,22].

Elastin is a major ECM protein in the dermis that provides elasticity and resilience, allowing the skin to stretch and recover without permanent deformation [23]. Elastic fibers account for approximately 2–4% of skin protein content and contribute to skin elasticity, resilience, and mobility through their well-organized structure [24]. After a skin injury, elastin regeneration in a scar is minimal, contributing to the reduced elasticity and functional resilience of healed skin [24]. In addition to its mechanical role, elastin modulates cell migration, proliferation, ECM synthesis, and wound contraction, thereby improving the quality of regenerated tissue [5,25]. Furthermore, collagen–elastin composite scaffolds support epithelial and dermal cell growth, promote elastic fiber formation, and restore skin elasticity [23].

In the present study, we introduce TissueDerm Graft^®^, a novel collagen-based dermal substitute designed to improve both functional and aesthetic outcomes in skin graft procedures by providing a stable structure that supports improved tissue regeneration. The wound healing efficacy of TissueDerm Graft^®^ was compared to that of Matriderm^®^, a single-stage dermal substitute containing bovine collagen types 1, 3, 5 and elastin hydrolysate. Among the collagen-based dermal substitutes, Matriderm^®^ is a clinically established dermal substitute for one-stage reconstruction and has been extensively investigated for its clinical applications [5,26]. Therefore, Matriderm^®^ is widely adopted as a benchmark material to evaluate various collagen-based dermal substitutes. Accordingly, Matriderm^®^ was selected as the comparator in the present study to evaluate the regenerative performance of TissueDerm Graft^®^ under clinically relevant conditions. In the present study, the histological characteristics and mechanical properties of TissueDerm Graft^®^ were compared to those of Matriderm^®^. A full-thickness skin defect was induced in minipigs, and TissueDerm Graft^®^ and Matriderm^®^ were implanted with a split-thickness skin graft (STSG). The wound healing efficacy of TissueDerm Graft^®^ was evaluated by analyzing the visual outcomes, histological analysis, inflammatory responses, and scar formation.

## 2. Materials and Methods

### 2.1. Fabrication of TissueDerm Graft^®^

To fabricate the TissueDerm Graft^®^, atelocollagen was extracted from sliced porcine skin by soaking it in a mild acidified pepsin treatment [27]. Distilled water was used to manufacture 1.8% (*w*/*v*) atelocollagen (MSbio, Seongnam-si, Republic of Korea), and the collagen solution was adjusted to pH 3.2 with 1 N HCL (Ducksan, Seoul, Republic of Korea). Glacial acetic acid (Daejung, Siheung-si, Republic of Korea) was added to produce 1.5% (*w*/*v*) acetic acid in the solution. The solution was centrifuged at 7000 RPM for 10 min and aliquoted in a square tray for freezing at −80 °C. After 24 h, the solution was freeze-dried at −72 °C and vaporized for 48 h. To neutralize and discard the salts, such as acetic acid, in the atelocollagen, the freeze-dried collagen was washed four times for 15 min with anhydrous alcohol (Daejung, Siheung-si, Republic of Korea) and 0.5 M NaOH (Daejung, Siheung-si, Republic of Korea) in 70% EtOH (DUKSAN, Seoul, Republic of Korea). To remove the remaining NaOH, the collagen sheet was sequentially washed four times for 15 min each with 50% and 30% ethanol and distilled water. After washing, the collagen was cooled to −70 °C for 12 h and freeze-dried for 24 h to obtain a TissueDerm Graft^®^. The alignment of collagen fibrils and pore distribution of TissueDerm Graft^®^ were compared to those of Matriderm^®^ (MedSkin Solutions Dr. Suwelack AG, Billerbeck, Germany) by scanning electron microscope (SEM, Merlin, Zeiss, Oberkochen, Germany).

### 2.2. Tensile Testing

The mechanical properties of TissueDerm Graft^®^ and Matriderm^®^ were evaluated using a universal testing machine with a 50 kgf capacity load cell (TESTONE Co., Ltd., Siheung-si, Republic of Korea). Among the various mechanical assessments, a tensile strength test was selected for the analysis and performed in triplicate for each group. For the tensile strength test, all the samples were cut into standardized dimensions of 45 mm × 45 mm × 1 mm. Each sample was mounted flat and securely clamped between the grips of the testing jig, maintaining a grip-to-grip distance of 30 mm. Tensile testing was conducted at a crosshead speed of 100 mm/min. The load–displacement data were continuously recorded until specimen failure. The mean value of tensile strength test was used for analysis.

### 2.3. Histological Properties of TissueDerm Graft^®^

The histological properties of TissueDerm Graft^®^ were examined to confirm its components. All samples were fixed by immersion in 4% paraformaldehyde for one day. The fixed samples were paraffin-embedded and sliced to 4 μm thickness for analysis. Hematoxylin & eosin (H&E) and Verhoff Van Gieson (VVG) staining were performed to evaluate the histological characteristics and ECM components of TissueDerm Graft^®^ compared to those of Matriderm^®^. Images were obtained using an IX73 microscope (Olympus, Tokyo, Japan), and a quantitative analysis of elastin was performed. Immunofluorescence was performed to confirm the contents of collagen types 1 and 3 in the TissueDerm Graft^®^ compared to the Matriderm^®^ groups. The sections were deparaffinized with xylene (Ducksan, Seoul, Republic of Korea) and hydrated with graded EtOH (100–50% EtOH) (Ducksan, Seoul, Republic of Korea) and distilled water. The hydrated sections were treated with a pepsin solution (GBI Labs, Tacoma, WA, USA) for 10 min for epitope retrieval. BLOXALL^®^ Endogenous Blocking Solution (VECTOR Laboratories, Newark, CA, USA) was applied for 10 min to inhibit the peroxidase activity. Then, 5% normal horse serum (VECTOR Laboratories, Burlingame, CA, USA) was used to block the unspecified epitopes. Subsequently, the sections were incubated with rabbit anti-collagen type 1 (1:100; Proteintech, Cambridge, UK) and mouse anti-collagen type 3 (1:100; Proteintech, Cambridge, UK) antibodies at 4 °C overnight. After washing with 0.5% Tween20 (Junsei, Tokyo, Japan) in phosphate-buffered saline (PBS-T), the samples were incubated with Alexa 488-conjugated anti-rabbit IgG (1:200; CST, Danvers, MA, USA) or Alexa 555-conjugated anti-mouse IgG (1:200; CST, MA, USA) antibodies at room temperature for 2 h in the dark. The stained sections were dehydrated with graded EtOH and mounted with an anti-fade mounting solution. The images were captured using LSM700 confocal microscopy (Carl Zeiss, Oberkochen, Germany).

### 2.4. Co-Implantation of STSG and TissueDerm Graft^®^ in Skin Defect Model

All animal experiments in the present study were approved by the Institutional Animal Care and Use Committee of Yonsei University College of Medicine (IACUC 2022–0333; date: 23 January 2023 to 22 January 2025), in compliance with ARRIVE guidelines. Four minipigs (12 months old, 25 kg, female) were purchased from APURES Co., Ltd. (Pyeongtaek, Republic of Korea). After seven days of acclimatization, the minipigs were anesthetized with an intramuscular injection of 1 mg/kg alfaxalone (Jurox Inc., Kansas City, MO, USA) and continuous inhalational administration of 2–3% isoflurane (Ifran Solution; Hana Pharm, Seoul, Republic of Korea). They were divided into 4 experimental groups: (1) normal, unwounded skin (sham); (2) single implantation of STSG after full-thickness skin defect; (3) co-implantation of STSG and Matriderm^®^ after full-thickness skin defect; and (4) co-implantation of STSG and TissueDerm Graft^®^ after full-thickness skin defect. Briefly, to prepare the skin defect model, a 35 mm × 35 mm split-thickness skin graft (approximately 2 mm thick) was initially obtained manually using a dermatome. Then, an additional 4 mm thickness of dermal tissue was excised to manufacture the full-thickness skin defect model. After the operation, TissueDerm Graft^®^ or Matriderm^®^ was placed onto the skin defect, and the harvested STSG was immediately placed over the dermal substitute without meshing. After the operation, all experimental groups were sutured with a nonabsorbable nylon suture and a medical stapler (COVIDIEN, Mansfield, MA, USA) and draped using Ioban2 drape (3M, St. Paul, MN, USA). All groups in the present study were tested on six repeats (N = 6) from each minipig.

### 2.5. Modified Vancouver Scar Scale Score

For evaluation of visual outcomes after co-implantation of STSG and TissueDerm Graft^®^, the modified Vancouver scar scale (mVSS) score was measured. Four surgeons evaluated the vascularity and pigmentation, as described by the mVSS [28,29]. Vascularity was evaluated using four grades (0 = normal; 1 = pink; 2 = red; 3 = purple), and pigmentation of each group was evaluated using four grades (0 = normal; 1 = hypopigmentation; 2 = mixed pigmentation; 3 = hyperpigmentation). The mVSS scores were visualized and analyzed using GraphPad Prism 8 (GraphPad Software, LLC, San Diego, CA, USA).

### 2.6. Histological Analysis

The minipigs were euthanized at 2, 4, and 8 weeks postoperatively. The euthanasia was performed under anesthesia with an intramuscular injection of 1 mg/kg alfaxalone (Jurox Inc., MO, USA) followed by a KCl injection. The samples were surgically obtained after euthanasia, and the hypodermis was manually discarded. The samples were washed several times with 2% antibiotics and antimycotics (Thermo Fisher Scientific, Waltham, MA, USA) in PBS (WELGENE, Seoul, Republic of Korea), and fixed by immersion in 4% paraformaldehyde for 7 days. The fixed samples were trimmed for paraffin embedding, and the samples were sectioned into 4 μm thick sections for a histological analysis. H&E and Masson’s trichrome (MT) staining were performed to evaluate the histological characteristics of co-implantation of STSG and TissueDerm Graft^®^ after a full-thickness skin defect. The images were acquired using an IX73 microscope (Olympus, Tokyo, Japan). Both the wound healing area and neodermis thickness were manually measured using ImageJ Fiji software (version 2.35, National Institutes of Health) at 2, 4, and 8 weeks postoperatively, and the wound healing area was calculated in each group according to the following formula:

Wound Healing Area = Normal Area (Sham)-Scar Area.

### 2.7. Immunofluorescence

Immunofluorescence was performed to confirm the characteristics of regenerated tissues after co-implantation of STSG and TissueDerm Graft^®^ in the full-thickness skin defect model. The sections were deparaffinized with xylene (Ducksan, Seoul, Republic of Korea) and hydrated with 100–50% EtOH (Ducksan, Seoul, Republic of Korea) and distilled water. The hydrated sections were treated with a pepsin solution (E06-50, GBI Labs, WA, USA) for 10 min for epitope retrieval. BLOXALL^®^ Endogenous Blocking Solution (SP-6000-100, VECTOR Laboratories, CA, USA) was applied for 10 min to inhibit peroxidase activity. Then, 5% normal horse serum (S-2000-20; VECTOR Laboratories, Burlingame, CA, USA) was used to block the unspecified epitopes. Subsequently, the sections were incubated with rabbit anti-TNF-α (1:200; BS-0078R, bioss, Woburn, MA, USA), rabbit anti-IL-6 (1:200; BS-4587R, bioss, MA, USA), or rabbit anti-α-SMA (1:200; NBP1-30894, Novus biologicals, Centennial, CO, USA) antibodies at 4 °C overnight. Subsequently, the， sections were washed with 0.5% Tween 20 (Junsei, Tokyo, Japan) in PBS-T and incubated in the dark at room temperature for 2 h with Alexa 488-conjugated anti-rabbit IgG (1:200; 4412S, CST, MA, USA) or Alexa 647-conjugated anti-rabbit IgG (1:200; A-21245, Invitrogen, Waltham, MA, USA) antibodies. The stained sections were dehydrated with graded EtOH and mounted with Fluoroshield mounting solution containing DAPI (ab104139, Abcam, Cambridge, UK). The images were captured using a fluorescence microscope (Olympus, Tokyo, Japan), and the intensity of immunostaining was analyzed using ImageJ Fiji software (version 2.35, National Institutes of Health).

### 2.8. Statistical Analysis

All the quantitative data are presented as the mean ± standard deviation except for the mVSS scores. Statistical comparisons of quantitative data were performed using one-way analysis of variance (ANOVA) followed by Tukey’s multiple comparisons test. The mVSS scores were analyzed using the Kruskal–Wallis test followed by Dunn’s multiple comparisons test. All statistical analyses were performed with GraphPad Prism 8 (San Diego, CA, USA). Differences were considered statistically significant at ns (not significant), * *p* < 0.05, ** *p* < 0.01, *** *p* < 0.001, and **** *p* < 0.0001.

## 3. Results

### 3.1. Characteristics of TissueDerm Graft^®^

**Figure 1:**
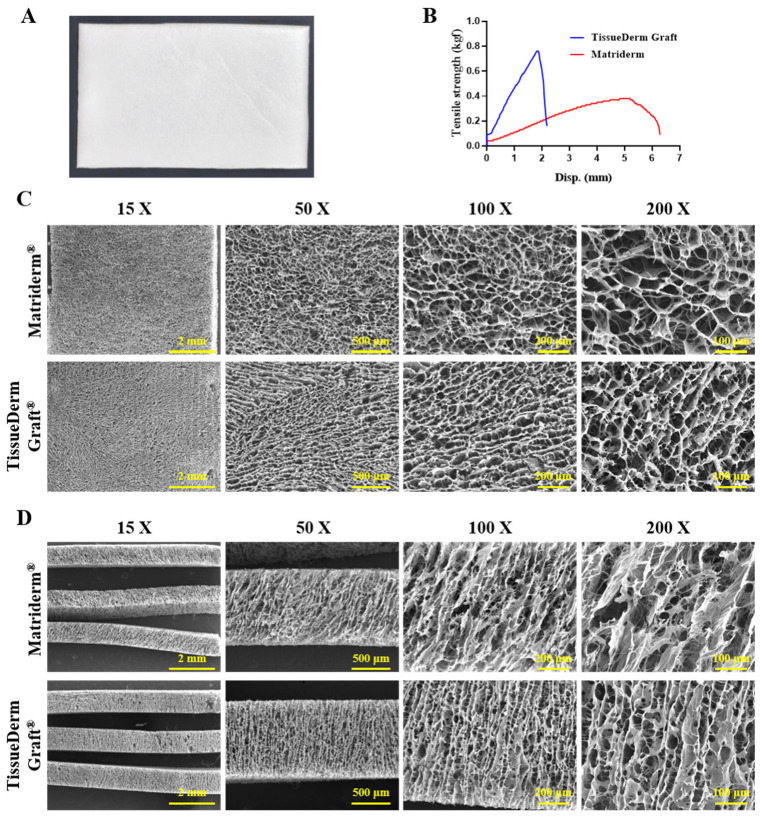
Characteristics of TissueDerm Graft^®^. (**A**) Morphology of TissueDerm Graft^®^. (**B**) Tensile strength tests for TissueDerm Graft^®^ and Matriderm^®^. (**C**) Representative SEM images of top surfaces of TissueDerm Graft^®^ and Matriderm^®^. (**D**) Representative SEM images of cross-sections of TissueDerm Graft^®^ and Matriderm^®^. Scale bars are shown in each panel.

The morphology of TissueDerm Graft^®^ is presented in Figure 1A and the mechanical properties of TissueDerm Graft^®^ and Matriderm^®^ are presented in Figure 1B. The maximum tensile strength of TissueDerm Graft^®^ was 0.76 kgf, whereas that of Matriderm^®^ was 0.36 kgf, demonstrating that the TissueDerm Graft^®^ exhibits approximately twice the tensile strength of that of Matriderm^®^. To analyze the structure of TissueDerm Graft^®^ compared to that of Matriderm^®^, the morphology of TissueDerm Graft^®^ and Matriderm^®^ were captured by SEM (Figure 1C,D). The structure of TissueDerm Graft^®^ and Matriderm^®^ were captured in sight of surface and cross-sectioned space. As a result, the collagen fibrils and pore distribution in the collagen sheets were homogeneously distributed and generally parallel. The pore distribution and porosity of TissueDerm Graft^®^ were more uniform than those of Matriderm^®^ based on the SEM in sight of surface. The surface pore diameter of TissueDerm Graft^®^ was 31–70 μm and the surface pore diameter of Matriderm^®^ was 56–170 μm. However, the cross-sectional pore diameter of TissueDerm Graft^®^ was 29–75 μm and that of Matriderm^®^ was 38–84 μm, indicating that the cross-sectional pore diameter of TissueDerm Graft^®^ was similar to that of Matriderm^®^. These results indicate that TissueDerm Graft^®^ has characteristics similar to those of Matriderm^®^.

### 3.2. Histological Analysis of TissueDerm Graft^®^

**Figure 2:**
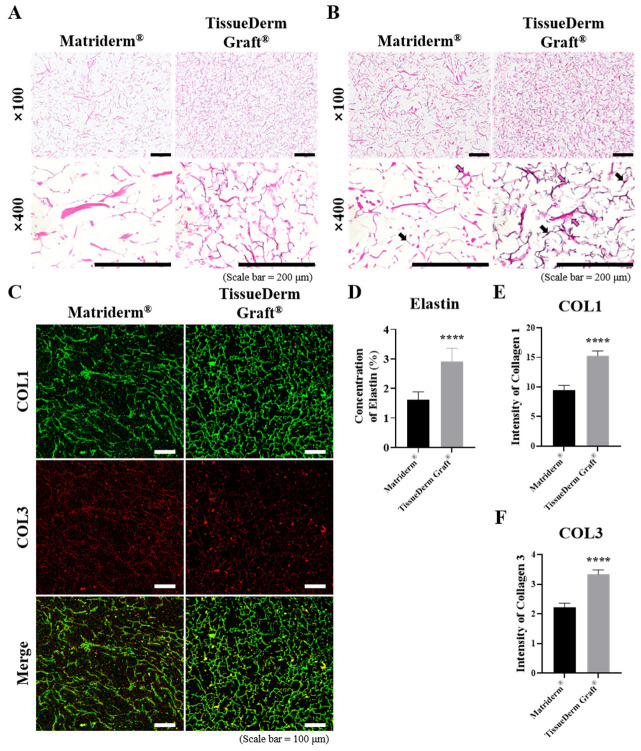
Histological analysis of TissueDerm Graft^®^. (**A**) Representative H&E images of TissueDerm Graft^®^ and Matriderm^®^ (scale bar = 200 μm). (**B**) Representative Verhoff Van Gieson (VVG) images of TissueDerm Graft^®^ and Matriderm^®^ (pink arrow: collagen fiber; black arrow: elastin fiber; scale bar = 200 μm). (**C**) Representative immunofluorescence images of COL1 (green) and COL3 (red) in TissueDerm Graft^®^ and Matriderm^®^ (scale bar = 100 μm). (**D**) Quantitative analysis of elastin. (**E**) Quantitative analysis of COL1. (**F**) Quantitative analysis of COL3. Statistical analysis was performed using one-way ANOVA (**** *p* < 0.0001).

The histological analysis confirmed the contents of TissueDerm Graft^®^. As a result, the representative images of H&E staining showed that the collagen fibril in TissueDerm Graft^®^ was relatively uniform compared to that of Matriderm^®^, and the density of collagen in TissueDerm Graft^®^ was higher than that of Matriderm^®^ (Figure 2A). Next, VVG staining was performed to evaluate the content of elastin in TissueDerm Graft^®^, whereas immunofluorescence was performed to evaluate the content of collagen types 1 and 3 in TissueDerm Graft^®^ compared to that of Matriderm^®^ (Figure 2B,C). The results showed that the content of collagen and elastin fibers was relatively higher in the TissueDerm Graft^®^ compared to that of Matriderm^®^. Moreover, the quantitative analysis demonstrated that the content of elastin in Matriderm^®^ and TissueDerm Graft^®^ was 1.62 and 2.89%, respectively (Figure 2D). In addition, the quantitative analysis of immunofluorescence showed that the content of collagen was significantly higher in TissueDerm Graft^®^ compared to that of Matriderm^®^. These results demonstrate that TissueDerm Graft^®^ has a higher and more uniform content of ECM compared to that of Matriderm^®^.

### 3.3. In Vivo Analysis via Modified Vancouver Scar Scale Score

**Figure 3:**
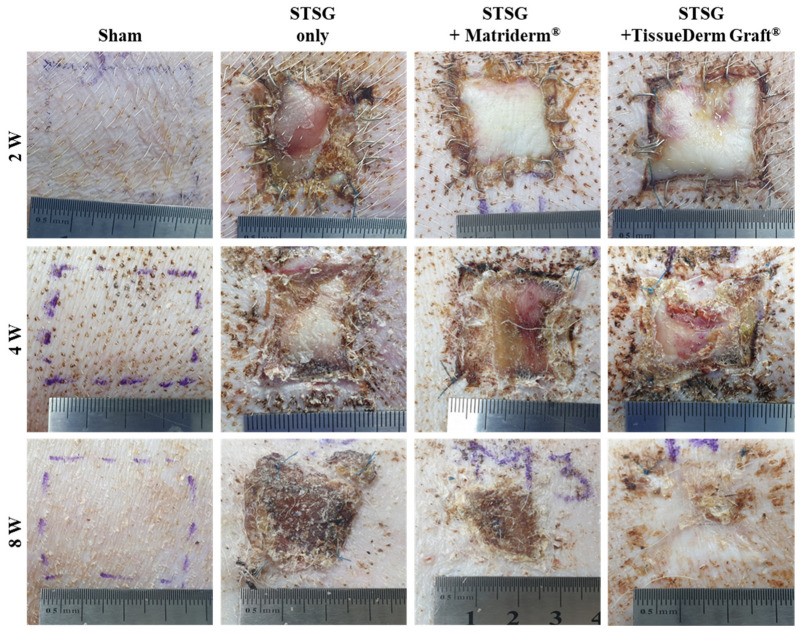
In vivo analysis of TissueDerm Graft^®^ in full-thickness skin defect model in minipigs. The visual outcomes were captured at 2, 4, and 8 weeks postoperatively.

**Figure 4:**
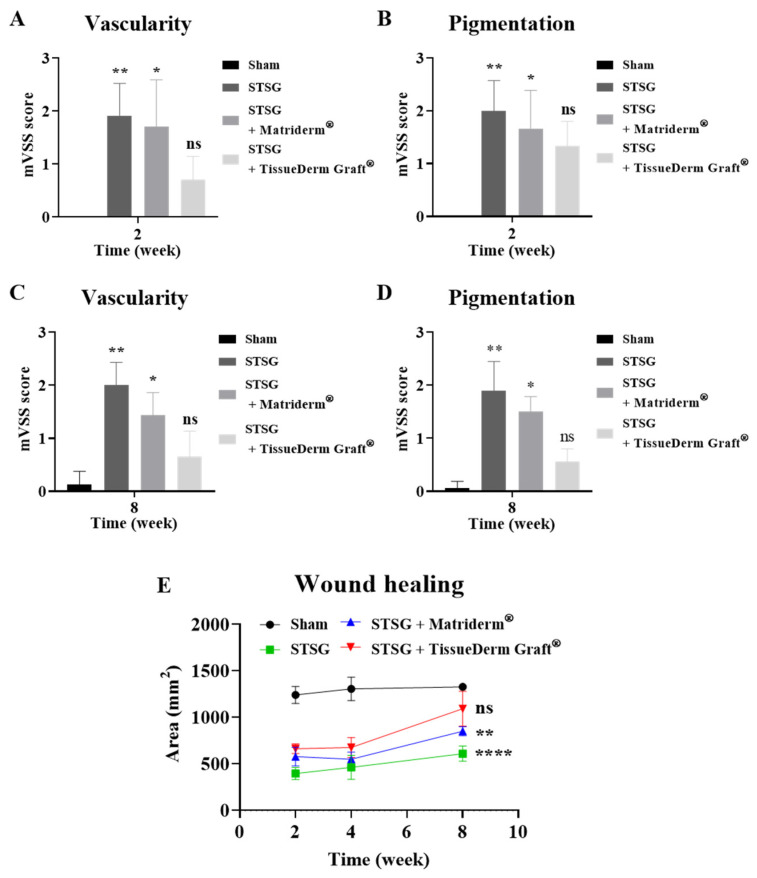
In vivo analysis via modified Vancouver scar scale and wound healing. (**A**) The vascularity was evaluated for visual outcomes at 2 weeks postoperatively using the mVSS score. (**B**) The pigmentation was evaluated for visual outcomes at 2 weeks postoperatively using the mVSS score. (**C**) The vascularity was evaluated for visual outcomes at 8 weeks postoperatively using the mVSS score. (**D**) The pigmentation was evaluated for visual outcomes at 8 weeks postoperatively using the mVSS score. The statistical analysis of mVSS scores was performed using the Kruskal–Wallis test followed by Dunn’s multiple comparisons test (ns, not significant; * *p* < 0.05; ** *p* < 0.01). (**E**) Quantitative analysis of wound healing postoperatively. Statistical analysis was performed using a one-way ANOVA (ns, not significant; ** *p* < 0.01; **** *p* < 0.0001).

An in vivo analysis was performed to demonstrate the properties of co-implantation of STSG and TissueDerm Graft^®^ in wound healing. The morphology of each group was captured at 2, 4, and 8 weeks postoperatively (Figure 3). The vascularity and pigmentation were evaluated using the mVSS score at 2 and 8 weeks postoperatively (Figure 4A–D). The vascularity and pigmentation significantly increased after a single implantation of STSG in a full-thickness skin defect. Conversely, the vascularity and pigmentation scores decreased after co-implantation of STSG and TissueDerm Graft^®^ or co-implantation of STSG and Matriderm^®^. Particularly, the vascularity and pigmentation scores showed that there was no significant difference between the sham group and co-implantation of STSG and TissueDerm Graft^®^ group. These results confirm that TissueDerm Graft^®^ has a superior effect on wound healing compared to that of Matriderm^®^, and that it can be utilized for clinical purposes in full-thickness skin defects.

### 3.4. Wound Healing

Wound healing was analyzed using ImageJ Fiji (Figure 4E). Wound healing was manually measured at 2, 4, and 8 weeks postoperatively. The area of initial skin was 1225 mm^2^, which enlarged to 1240.1 ± 91.6, 1306.1 ± 127.5, and 1328.0 ± 38.8 mm^2^ at 2, 4, and 8 weeks postoperatively following growth of minipigs, respectively. After the full-thickness skin defect, the area of the skin defect initially decreased and then recovered after STSG implantation. The area of skin defect after single implantation of STSG was 396.2 ± 66.0, 463.1 ± 128.1, and 609.0 ± 82.1 mm^2^ at 2, 4, and 8 weeks postoperatively, respectively. The area of skin defect after co-implantation of STSG and Matriderm^®^ was 578.2 ± 101.5, 548.6 ± 77.6, and 850.4 ± 48.9 mm^2^ at 2, 4, and 8 weeks postoperatively, respectively. The area of skin defect after co-implantation of STSG and TissueDerm Graft^®^ was 663.2 ± 51.8, 676.0 ± 106.8, and 1092.3 ± 188.5 mm^2^ at 2, 4, and 8 weeks postoperatively, respectively. The quantitative analysis of wound healing showed that there was no significant difference between the sham group and co-implantation of STSG and TissueDerm Graft^®^ group. These results indicate that TissueDerm Graft^®^ increases the performance of STSG and wound healing in full-thickness skin defects and that TissueDerm Graft^®^ has a superior effect on wound healing compared to Matriderm^®^.

### 3.5. Histological Analysis

**Figure 5:**
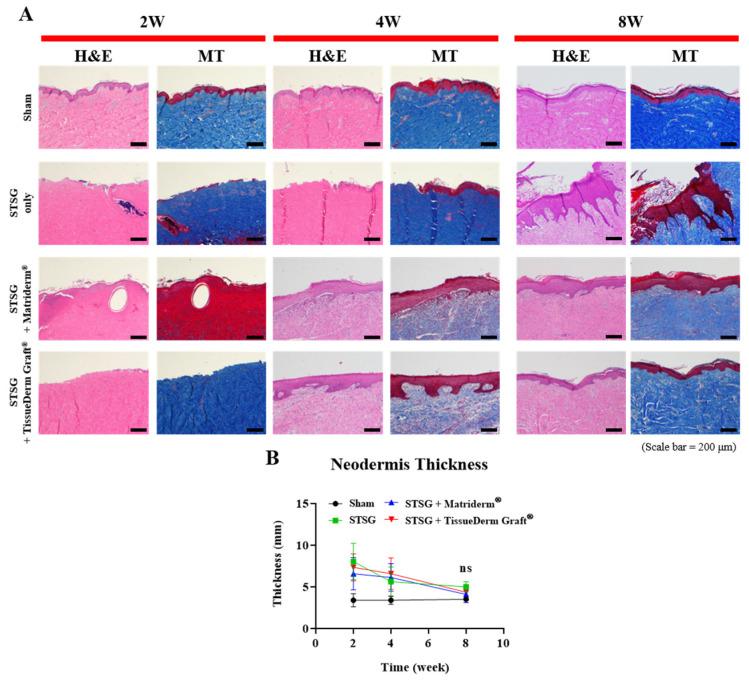
Histological analysis. (**A**) Representative images of hematoxylin & eosin and Masson’s trichrome staining (scale bar = 200 μm). The epidermal and dermal tissues were homogeneously regenerated after co-implantation of STSG and TissueDerm Graft^®^ or Matriderm^®^ in the full-thickness skin defect model. (**B**) Quantitative analysis of neodermis thickness. Statistical analysis was performed using one-way ANOVA (ns, not significant).

A histological analysis was performed to confirm wound healing after co-implantation of STSG and TissueDerm Graft^®^ in the full-thickness skin defect model. All groups were analyzed at 2, 4, and 8 weeks postoperatively, and the morphology was observed by H&E and MT staining, which showed that the epidermis and dermis of the sham group were well arranged in all experimental periods (Figure 5A). However, the epidermis of a single implantation of the STSG group was barely observed at 2 weeks postoperatively. It was observed at 4 weeks after single implantation of STSG and the skin regenerated with a heterogeneous epidermis at 8 weeks postoperatively.

Co-implantation of STSG and TissueDerm Graft^®^ and co-implantation of STSG with Matriderm^®^ improved the outcomes of STSG in full-thickness skin defects. 4 and 8 weeks after co-implantation, the dermis and epidermis were homogenously regenerated compared to the single implantation of STSG.

Next, the neodermis thickness was analyzed to determine the healing stage after co-implantation of STSG and TissueDerm Graft^®^ in a full-thickness skin defect (Figure 5B). The thickness of the neodermis was measured from the skin defect to the basement of the epidermis using ImageJ software. The neodermis thickness after single implantation of STSG was 8.03 ± 1.99 mm, 5.62 ± 1.60, and 5.00 ± 0.56 mm at 2, 4, and 8 weeks postoperatively, respectively. The neodermis thickness after co-implantation of STSG and Matriderm^®^ was 6.57 ± 1.77, 6.12 ± 1.52, and 4.10 ± 0.91 mm at 2, 4, and 8 weeks postoperatively. The neodermis thickness after co-implantation of STSG and TissueDerm Graft^®^ was 7.34 ± 1.48, 6.56 ± 1.73, and 4.35 ± 0.69 mm at 2, 4, and 8 weeks postoperatively. The statistical analysis showed a significant difference in the neodermis thickness between the single implantation of STSG and the co-implantation of STSG and collagen sheets. However, there was no significant difference between the TissueDerm Graft^®^ and Matriderm^®^. In the present study, the neodermis thickness was reduced by the co-implantation of STSG and collagen sheets. These findings indicate that TissueDerm Graft^®^ and Matriderm^®^ exhibited comparable neodermis thickness throughout the healing period.

### 3.6. Inflammatory Responses

**Figure 6:**
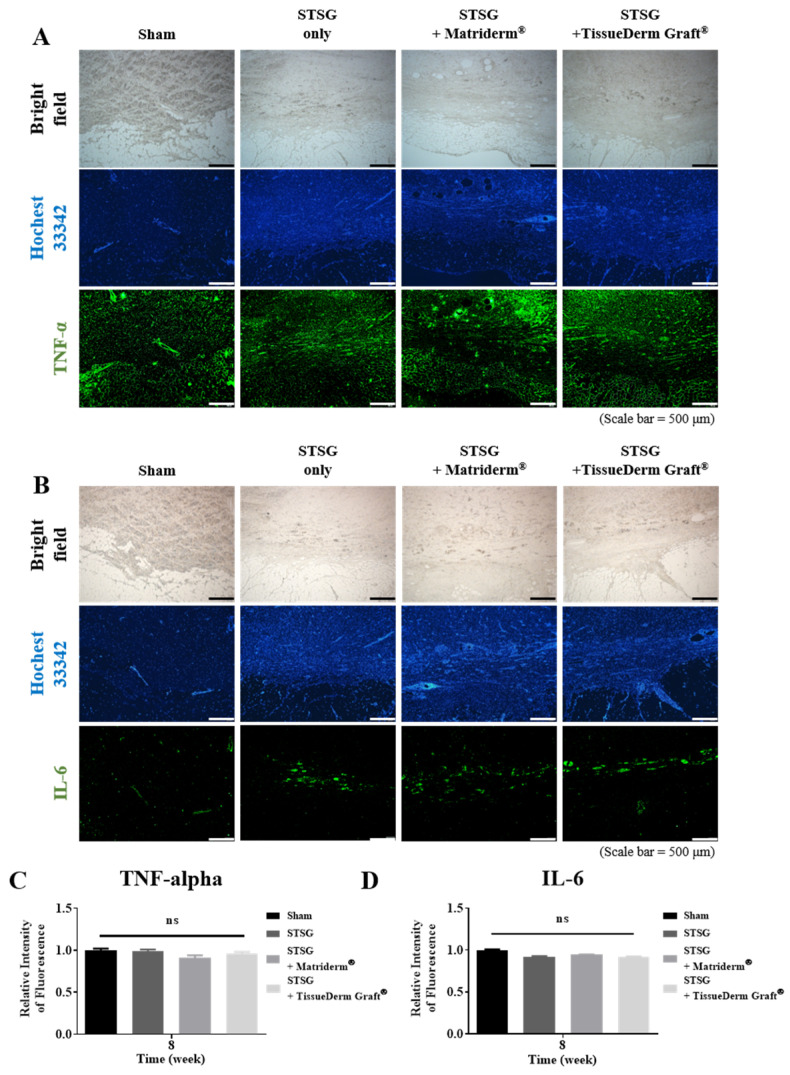
Inflammatory response. (**A**) Representative immunofluorescence images of TNF-α (scale bar = 500 μm). (**B**) Representative immunofluorescence images of IL-6 (scale bar = 500 μm). (**C**) Quantitative analysis of intensity of TNF-α. (**D**) Quantitative analysis of intensity of IL-6. Statistical analysis was performed using one-way ANOVA (ns, not significant).

An inflammatory response is a physiological reaction that can occur owing to injury, microbes, or foreign bodies, and it rarely occurs after the implantation of an allogenic skin graft. Here, immunofluorescence was performed to confirm the inflammatory response after co-implantation of STSG and TissueDerm Graft^®^ at 8 weeks postoperatively (Figure 6A,B). In the present study, the expression of TNF-α and IL-6 were evaluated to confirm the inflammatory response. The quantitative analysis revealed that there was no significant difference in the expression of TNF-α or IL-6 between the single implantation of STSG and co-implantation of collagen sheets (Figure 6C,D). These results indicate that TissueDerm Graft^®^ does not induce an excessive inflammatory response, similar to that of Matriderm^®^.

### 3.7. Scar Formation

**Figure 7:**
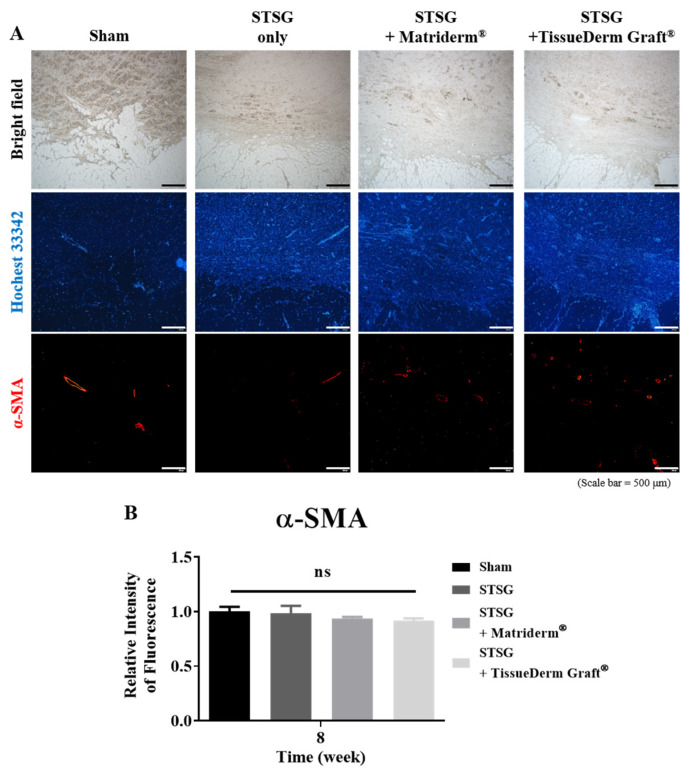
Scar formation. (**A**) Representative immunofluorescence images of α-SMA (scale bar = 500 μm). (**B**) Quantitative analysis of intensity of α-SMA. Statistical analysis was performed using one-way ANOVA (ns, not significant).

Following the results of inflammatory response, immunofluorescence was performed to demonstrate the degree of scar formation after co-implantation of STSG and TissueDerm Graft^®^ 8 weeks postoperatively (Figure 7A). Scar formation can be upregulated during wound healing via the recruitment of inflammatory cells, including macrophages, mast cells, and neutrophils. In the present study, the expression of α-SMA, a well-known marker of scar formation, was observed by immunofluorescence. As a result, its expression was not increased after a single implantation of STSG or co-implantation of STSG and collagen sheets (Figure 7B). Therefore, these results suggest that implantation of TissueDerm Graft^®^ does not induce scar formation, similar to Matriderm^®^.

## 4. Discussion

This study was conducted to evaluate the wound healing efficacy of TissueDerm Graft^®^ using a full-thickness skin defect model in minipigs. In the first phase, the structure and physical properties of TissueDerm Graft^®^ were comprehensively characterized, and its features were compared with those of Matriderm^®^, a dermal substitute commonly used in clinical practice. After co-implantation of STSG and TissueDerm Graft^®^ in a full-thickness skin defect model, the visual outcomes were quantified based on the mVSS score and a histological analysis was performed to evaluate the tissue regeneration, inflammatory response, and scar formation compared to Matriderm^®^. In this study, we provide insights into the potential application and effectiveness of TissueDerm Graft^®^ for promoting functional and aesthetic wound healing by comparing it with established clinical standards.

Understanding the structural and mechanical properties of dermal replacement materials is critical because these factors directly influence wound healing outcomes, including tissue regeneration, control of the inflammatory response, and minimization of scar formation [30,31]. In addition to providing structural stability during implantation and handling, sufficient mechanical strength may help maintain the integrity of dermal substitutes under physiological loading conditions. In the present study, TissueDerm Graft^®^ exhibited approximately two-fold higher tensile strength than Matriderm^®^, indicating the enhanced mechanical strength of the graft. In addition to mechanical strength, pore distribution is an important determinant of the biological performance of collagen-based dermal substitutes. Previous studies have demonstrated that interconnected pores with an appropriate size and uniform distribution facilitate fibroblast infiltration, vascular ingrowth, nutrient diffusion, and extracellular matrix remodeling, thereby promoting the successful integration of dermal substitutes and STSG engraftment [32,33]. In the present study, TissueDerm Graft^®^ exhibited a more homogeneous pore distribution than Matriderm^®^ while maintaining a comparable cross-sectional pore diameter, suggesting that its porous architecture may provide a favorable microenvironment for tissue regeneration and graft integration.

Wound healing is an evolutionarily conserved and complex process that aims to restore the skin barrier. This process is typically divided into four overlapping phases: hemostasis, inflammation, proliferation, and remodeling. Each phase involves the coordinated activity of various cell types, including keratinocytes, fibroblasts, endothelial cells, macrophages, and platelets. In previous studies, collagen-based dermal substitutes have been reported to provide a favorable extracellular matrix for cellular infiltration, vascularization and dermal regeneration, thereby improving wound healing outcomes [34,35,36].

TissueDerm Graft^®^, compared with Matriderm^®^, exhibits a structure with aligned collagen fibers, which may provide a favorable microenvironment for cell migration [37,38]. Such aligned scaffolds guide cells through contact, which has been reported to facilitate organized cellular alignment and ECM remodeling in previous scaffold studies [39,40,41]. Additionally, the SEM and immunofluorescence staining images show that TissueDerm Graft^®^ exhibits a denser collagen network than Matriderm^®^. Furthermore, it is confirmed that TissueDerm Graft^®^ contains higher elastin content than Matriderm^®^. The elastin content is comparable to that reported for normal human skin (approximately 2–4%) [42]. These structural characteristics may contribute to the favorable wound healing outcomes observed in the present study. However, further studies investigating early-stage graft survival parameters, in vivo degradation kinetics, cytotoxicity and fibroblast infiltration using fibroblasts or keratinocytes are required to fully elucidate the biological mechanisms underlying its regenerative potential.

The mVSS score was used to evaluate macroscopic wound healing, particularly vascularity and pigmentation, at early (2 weeks) and intermediate (8 weeks) stages postoperatively. Vascularity and pigmentation were selected as important macroscopic parameters because vascularization during early wound healing plays a critical role in oxygen and nutrient supply, dermal substitute integration, and STSG survival [43,44]. In contrast, pigmentation represents clinically relevant aesthetic outcomes associated with skin regeneration and scar remodeling [45]. The mVSS score is a well-established scar assessment tool that includes vascularity and pigmentation as key evaluation parameters [46]. It has acceptable internal consistency and inter-observer reliability in the evaluation of skin graft scars, including facial graft scars [9]. To improve the reliability of the assessment, vascularity and pigmentation were independently evaluated by a panel of experienced clinical plastic surgeons using the mVSS score. The vascularity and pigmentation were significantly higher after a single implantation of the STSG compared to those of the sham group. In contrast, both parameters were significantly reduced after co-implantation of STSG and Matriderm^®^ and co-implantation of STSG and TissueDerm Graft^®^ compared to after the single implantation of STSG. Notably, the vascularity and pigmentation were significantly lower after co-implantation of STSG and TissueDerm Graft^®^ compared to Matriderm^®^. Although the vascularity was macroscopically evaluated using the mVSS score in the present study, future studies incorporating an objective histological evaluation of vascularization, such as CD31 immunostaining, will provide further insight into the regenerative mechanisms of TissueDerm Graft^®^.

A histological analysis and immunostaining were performed to evaluate the inflammatory response and scar formation. Although no statistically significant differences in inflammatory markers were observed among the groups, the TissueDerm Graft^®^ showed favorable wound healing without inducing excessive inflammatory responses or excessive scar formation. In the early stage of wound healing, although no additional materials were implanted in the full-thickness skin defect, the neodermis thickness was the highest after the single implantation of STSG and decreased with co-implantation of Matriderm^®^ or TissueDerm Graft^®^. It was expected that Matriderm^®^ and TissueDerm Graft^®^ would regulate the initial inflammatory response; hence, the recruitment of immune cells, such as macrophages and neutrophils, as well as swelling in the skin defects, was suppressed. In future studies, it is expected that the inflammatory response will show a significant difference in the early stages of wound healing.

These findings suggest that TissueDerm Graft^®^ may serve as an alternative collagen-based dermal substitute with comparable regenerative performance to Matriderm^®^. TissueDerm Graft^®^ is derived from porcine collagen, whereas Matriderm^®^ is derived from bovine collagen. Although both materials have been widely used in regenerative medicine, their differences in animal origin may influence their clinical acceptance depending on regional, cultural, or patient-specific considerations.

## 5. Conclusions

In the present study, the dermal regenerative properties of TissueDerm Graft^®^ were evaluated for up to 8 weeks, which may not adequately reflect the long-term effects of the test material in wound healing. Further studies are needed to investigate the biological mechanisms of dermal substitutes, such as fibroblast infiltration, cytotoxicity, vascularization and in vivo degradation. In conclusion, TissueDerm Graft^®^ demonstrated favorable wound healing outcomes associated with its structural characteristics and extracellular matrix composition in the full-thickness skin defect model. Although additional studies are required to investigate its long-term safety, biological mechanisms, and clinical performance, the present findings suggest that TissueDerm Graft^®^ has potential as a collagen-based dermal substitute for skin regeneration.

## Data Availability

The data presented in this study are available on request from the corresponding author due to reasons of sensitivity.

## Abbreviations

The following abbreviations are used in this manuscript:

STSG	Split-thickness skin graft
FTSG	Full-thickness skin graft
mVSS	modified Vancouver scar scale
ECM	extracellular matrix
